# The Role of an Herbal Preparation in Enhancing the Efficacy of Basic Therapy in Patients with Periodontitis: Evaluation of Clinical, Microbiological and Cytomorphometric Parameters

**DOI:** 10.3390/ph19040586

**Published:** 2026-04-07

**Authors:** Ivana Stanković, Radmila Obradović, Ana Pejčić, Dušanka Kitić, Milica Petrović, Boško Toljić, Marija Bradić-Vasić, Katarina Živadinović

**Affiliations:** 1 Department of Periodontology and Oral Medicine, Dental Clinic, Faculty of Medicine, University of Niš, dr Zorana Djinjdjica Blvd 82, 18000 Niš, Serbia; 2 Department of Pharmacy, Faculty of Medicine, University of Niš, dr Zorana Djinjdjica Blvd 82, 18000 Niš, Serbia; dusanka.kitic@medfak.ni.ac.rs; 3 Department of General and Oral Physiology, School of Dental Medicine, University of Belgrade, dr Subotica 8, 11000 Belgrade, Serbia; bosko.toljic@stomf.bg.ac.rs; 4 Doctoral Studies, Department of Periodontology and Oral Medicine, Faculty of Medicine, University of Niš, dr Zorana Djinjdjica Blvd 82, 18000 Niš, Serbia

**Keywords:** periodontitis, periodontopathogens, clinical parameters, phytotherapy, basic therapy

## Abstract

**Objectives**: Periodontitis represents an inflammatory condition affecting the supporting structures of teeth. Basic therapy relies on scaling and root planning, but often fails to eliminate subgingival pathogens. Phytotherapy has emerged as an adjunct due to its antimicrobial, anti-inflammatory, antioxidant, and tissue-protective properties. The aim of this study was to evaluate the effects of the phytotherapeutic product Propoherb G^®^ as an adjunct to mechanical periodontal therapy. **Methods**: This study included 90 systemically healthy participants divided into three groups: control, basic therapy, and basic therapy + Propoherb G^®^. Periodontal clinical parameters were assessed, alongside the presence of periodontopathogens (*A. actinomycetemcomitans*, *P. gingivalis*, and *T. denticola*) using PCR and cytomorphometric analysis of gingival cells at baseline, first day, and eleven days after therapy. **Results**: Results showed significant improvements in periodontal clinical parameters in both treatment groups, with the most pronounced effect observed in the Propoherb G^®^ group (*p* < 0.001). A marked reduction in periodontopathogenic bacteria was achieved, with Propoherb G^®^ demonstrating sustained elimination of *P. gingivalis* and *T. denticola*, and a significant reduction of *A. actinomycetemcomitans* compared to standard therapy alone (*p* < 0.001). The control group showed no significant changes. Cytomorphometric analysis showed a significant decrease in all measured cell parameters after therapy (*p* < 0.001) in the group with Propoherb G^®^ preparation. **Conclusions**: The adjunctive use of Propoherb G^®^ enhances the clinical, microbiological and cytomorphometric outcomes of basic therapy. These findings support the potential of phytotherapy as a safe and effective supplement to basic treatment, although further studies with larger sample sizes and longer follow-up are necessary to standardize protocols and optimize clinical application.

## 1. Introduction

Periodontal diseases represent one of the most persistent oral health problems, with epidemiological findings indicating their presence even among ancient populations. Over time, therapeutic approaches have undergone significant changes, reflecting continuous advancements in dental science. In recent decades, progress in diagnostic techniques and treatment strategies has significantly influenced the current understanding and management of periodontal conditions, particularly in relation to predisposing and risk factors [1,2].

Periodontitis is a chronic, multifactorial inflammatory condition that affects the supporting structures of the teeth, including the gingiva, periodontal ligament, cementum, and alveolar bone. If left untreated, it represents a leading cause of tooth loss and may negatively impact systemic health [3,4,5,6]. The disease develops through a complex interaction between subgingival microbial biofilms and the host immune-inflammatory response. Key pathogens such as *Aggregatibacter actinomycetemcomitans* (*A.a.*), *Porphyromonas gingivalis* (*P.g.*), *Prevotella intermedia* (*P.i.*) and *Treponema denticola* (*T.d.*) contribute to disease onset and progression, while host responses, including pro-inflammatory cytokine release and reactive oxygen species production, exacerbate tissue destruction [3,7,8,9,10].

Basic periodontal therapy primarily relies on mechanical debridement, particularly scaling and root planning (SRP), combined with optimal oral hygiene [11]. Although SRP is effective, it often fails to eliminate all subgingival pathogens, particularly those residing in dentinal tubules, soft tissues, or root surface irregularities [7,11]. Adjunctive use of antimicrobial agents, such as chlorhexidine, triclosan, and cetylpyridinium chloride, has been employed to reduce bacterial invasion and prevent recolonization. However, their long-term use can result in adverse effects, including tooth staining, taste disturbances, mucosal irritation, and increased risk of bacterial resistance. These limitations have prompted the search for safer, more biocompatible alternatives [8,12,13,14,15].

Phytotherapy, the use of medicinal plants or plant-derived compounds, has emerged as a promising adjunctive or alternative approach in periodontal care [2,4,5,6]. Herbal agents possess biological activities, including antimicrobial, anti-inflammatory, antioxidant, antiseptic, and anti-collagenase properties. Bioactive constituents such as polyphenols, glycosides, tannins, essential oils, vitamins, and mineral salts contribute to the modulation of host inflammatory responses, reduction of oxidative stress, and inhibition of microbial growth [2,16,17,18]. Natural compounds are generally well tolerated, cost-effective, and safe, making them attractive alternatives to conventional chemical agents. The growing global use of herbal medicines, coupled with their favorable safety profile, highlights their potential role in improving periodontal treatment outcomes. Current evidence suggests that phytotherapeutic agents can support both surgical and non-surgical periodontal therapies by reducing inflammation, controlling biofilm formation, and promoting tissue regeneration. Nonetheless, further research in larger populations is required to standardize formulations, optimize dosages, and validate clinical efficacy [4,6,16,17,18,19,20,21].

Cytomorphometry is a technique of exfoliative cytology that enables the microscopic analysis of desquamated epithelial cells of the skin and mucous membranes. As a diagnostic method, it was introduced in the mid-20th century when Georgios Papanikolaou first applied it in the detection of malignant changes. This method provides a simple, rapid, painless, and non-invasive approach, which is why it is frequently used in the assessment of alterations in the oral mucosa [22].

### Aim of the Study

Due to the increasing popularity and potential benefits of herbal preparations, the aim of this study was to evaluate the effects of the phytotherapeutic product Propoherb G^®^ (Institute for Medicinal Plant Research “dr Josif Pančić”, Belgrade, Serbia) as an adjunct to basic periodontal therapy. The study assessed its impact on clinical periodontal parameters, including the plaque index (PLI), the gingival bleeding on probing (BOP) and clinical attachment level (CAL), as well as the presence of anaerobic bacteria (*Aggregatibacter actinomycetemcomitans*, *Porphyromonas gingivalis*, and *Treponema denticola*) and the cytomorphometric characteristics of gingival cells in patients with chronic periodontitis.

## 2. Results

A total of 90 systemically healthy subjects participated in the study. Table 1 presents the gender and age distribution of participants with chronic periodontitis, divided into two study groups according to the applied therapy: basic therapy (Test 1) and basic therapy + Propoherb G^®^ (Test 2), as well as a control group consisting of subjects with clinically healthy periodontium. Each group included 30 participants. In all examined groups, females were predominant, with the highest proportion observed in the Propoherb G^®^ group (80%), while the control group showed an even higher representation of females (90%), although without statistical significance (*p* = 0.090).

The average age of participants in the treatment groups ranged from 39.70 ± 7.14 to 43.50 ± 5.65 years, while the average age in the control group was significantly lower, at 26.47 ± 1.85 years. Analysis of variance (ANOVA) revealed a statistically significant difference in age among the groups (*p* < 0.001). Further post hoc Bonferroni analysis showed that the treatment groups had statistically significantly older participants compared to the control group: basic therapy (*p* < 0.001) and basic therapy + Propoherb G^®^ (*p* < 0.001).

### 2.1. Analysis of Clinical Parameters

At baseline (Table 2), the therapeutic groups showed similar values of the plaque index (PLI) (approximately 1.8–1.9), whereas the control group had significantly lower plaque index values (1.28), which was statistically confirmed (*p* < 0.001, Kruskal–Wallis’s test). Further post hoc analysis revealed that the PLI values were significantly higher in all therapeutic groups compared with the control group (*p* < 0.001, Mann–Whitney test). Comparative analysis among the groups after therapy demonstrated significant differences in treatment effects (Kruskal–Wallis’s test, *p* < 0.001). The lowest plaque index after therapy was recorded in the group receiving basic therapy with the addition of Propoherb G^®^ (0.19 ± 0.25), indicating a strong effect of this adjunctive treatment. The group treated with basic therapy alone had a PLI of 0.83 ± 0.37, which represents a significant improvement compared to baseline, although the effect was less pronounced than in the group receiving additional therapy. The control group showed no changes, and the plaque index remained the same (1.28 ± 0.50). Statistically significant differences were confirmed between the therapeutic groups and the control group, as well as between the basic therapy group and the group receiving Propoherb G^®^. After the applied therapy, a significant decrease in PLI was observed in all therapeutic groups (*p* < 0.001, Wilcoxon test), while no change was recorded in the control group (PLI remained 1.28).

Table 3 presents the results of the bleeding on probing index (BOP) before and after therapy for each study group. At baseline, the treatment groups showed almost identical BOP values, indicating the presence of moderate gingival inflammation, while in the control group the value was zero, which was statistically significant compared to the treatment groups (*p* < 0.001; Kruskal–Wallis’s test). After the applied therapeutic protocols, a significant reduction in the BOP index was observed in the examined groups. A pronounced decrease in BOP was recorded in the group receiving basic therapy combined with Propoherb G^®^ (0.16 ± 0.22), which was statistically significant compared to the basic therapy group (0.99 ± 0.36) (*p* < 0.001; Mann–Whitney test), as well as compared to the control group (0.0 ± 0.0) (*p* < 0.001; Mann–Whitney test). Within-group comparisons showed statistically significant improvements in BOP in both therapeutic groups after treatment (*p* < 0.001, Wilcoxon test).

Table 4 shows the CAL values before and after the applied therapy in the examined groups. Before therapy, all treated groups (basic therapy, basic therapy + Propoherb G^®^) had similar CAL values (around 3), while the control group had a value of 0.8 ± 0.33, which was statistically significant (*p* < 0.001; Kruskal–Wallis’s test). Statistical analysis revealed significant differences among the groups after therapy as well (*p* < 0.001; Kruskal–Wallis’s test). Post hoc testing confirmed that the therapeutic groups differed significantly from the control group (*p* < 0.001; Mann–Whitney test), as well as that the Propoherb G^®^ group was significantly more effective compared to basic therapy (*p* < 0.001; Mann–Whitney test). After therapy, a statistically significant reduction in CAL values was observed in both treated groups (*p* < 0.001, Wilcoxon test), with the most pronounced improvement recorded in the group that used Propoherb G^®^ in addition to basic therapy (2.14 ± 0.26).

### 2.2. Analysis of Microbiological Parameters

The results presented in Table 5 demonstrate the effectiveness of the therapeutic approaches in reducing the presence of *Porphyromonas gingivalis* in the participants. In the group receiving only basic therapy, a significant change was observed during the study period (Q = 18.727, *p* < 0.001). Positivity decreased significantly from 60.70% before therapy to 23.30% on the first day after therapy (*p* = 0.001) but increased again to 53.30% on the eleventh day (*p* = 0.004), indicating a temporary effect and a possible recurrence of infection. In participants who received basic therapy combined with Propoherb G^®^**,** positivity significantly decreased from 66.70% before therapy to 46.60% on the first day, and by the eleventh day the bacterium was eliminated (0%), representing a statistically significant and stable therapeutic effect (Q = 19.600, *p* < 0.001).

The post hoc McNemar test confirmed that the reduction in bacterial presence was significant between pre-therapy and the first day (*p* = 0.001), pre-therapy and the eleventh day (*p* < 0.001), as well as between the first and eleventh day (*p* < 0.001). The control group showed no changes throughout the study period, remaining at 10% positivity. Intergroup differences were significant before therapy (*p* = 0.010) and on the eleventh day (*p* < 0.001). The best outcome was observed in the group treated with Propoherb G^®^, confirming that the addition of this phytotherapeutic agent to basic therapy enables the most complete and lasting elimination of *Porphyromonas gingivalis*.

Within the basic therapy group, Cochran’s Q test (Q = 9.333, *p* = 0.009) demonstrated a statistically significant difference in the presence of *Aggregatibacter actinomycetemcomitans* across the three time points (pre-therapy, 1 day after therapy, and 11 days after therapy) (Table 6). Further analysis revealed that a significant reduction in bacterial presence was observed between the pre-therapy measurement and the measurement one day after therapy (*p* = 0.031, McNemar test). A statistically significant difference in the presence of *Aggregatibacter actinomycetemcomitans* was also observed across all three time points in the group receiving basic therapy combined with Propoherb G^®^ (Q = 29.778, *p* < 0.001). Post hoc McNemar testing indicated a significant reduction in bacterial presence between the pre-therapy measurement and the measurement on the 11th day (*p* < 0.001), as well as between the measurements taken on the 1st and 11th day (*p* < 0.001). A higher percentage of bacterial eradication was achieved in the group receiving basic therapy in combination with Propoherb G^®^. Statistically significant differences in *Aggregatibacter actinomycetemcomitans* presence between the therapeutic groups and the control were observed before therapy (χ^2^ = 25.173, *p* < 0.001), one day after therapy (χ^2^ = 11.670, *p* = 0.008), and on the 11th day after therapy (χ^2^ = 22.360, *p* < 0.001).

Table 7 shows the frequency of *Treponema denticola* positivity among participants in the study groups, before and after the application of different therapeutic approaches. In the group that received basic therapy, a significant change was observed over the study period (Q = 16.909, *p* < 0.001). A notable decrease in positivity was recorded one day after therapy (from 80,00% to 43.30%) (*p* = 0.001); however, there was a significant increase from 43.30% to 66.70% by the eleventh day (*p* = 0.016), indicating a short-term effect of the standard therapy. The group that received basic therapy combined with Propoherb G^®^ showed a better therapeutic response (Q = 32.095, *p* < 0.001). Positivity significantly decreased from 76.70% to 50% one day after therapy (*p* = 0.018) and then dropped drastically to 6.70% by the eleventh day (*p* < 0.001), including a significant difference between the first and eleventh day (*p* < 0.001), indicating a more pronounced and sustained effect. In the control group, the frequency of positivity remained unchanged (6.70%) across all time points, confirming that the observed effect in the other groups resulted from the applied therapy. Intergroup comparisons revealed significant differences before therapy (*p* < 0.001) compared to the control, on the first day after therapy (*p* = 0.002), and especially on the eleventh day (*p* < 0.001), further confirming the efficacy of the treatments, particularly in the group treated with Propoherb G^®^.

### 2.3. Analysis of Cytomorphometric Parameters

In the therapeutic groups, a statistically significant reduction in the nuclear area was observed after treatment (*p* < 0.001; Friedman test) (Table 8). The Wilcoxon test revealed significant differences between all measurement time points (baseline, day 1, and day 11 after therapy) within the therapeutic groups, with the greatest decrease recorded in the group receiving Propoherb G^®^ (from 92.61 ± 24.91 to 62.96 ± 13.63). No changes in values were observed over time in the control group. Comparisons between groups showed statistically significant differences across all three measurement points (baseline, day 1, and day 11) (*p* < 0.001, Kruskal–Wallis’s test), with particularly notable differences between the therapeutic groups and the control group (*p* < 0.001).

In the therapeutic groups, a statistically significant reduction in nuclear perimeter was observed after treatment (*p* < 0.001, repeated-measures ANOVA) (Table 9). Pairwise comparisons (*t*-test) showed that the differences were significant across all intervals: baseline vs. day 1 (a), baseline vs. day 11 (b), and day 1 vs. day 11 (c). This indicates a gradual but consistent decrease in nuclear perimeter as a result of the therapeutic effect. The group receiving Propoherb G^®^ as an adjunct demonstrated the greatest reduction in nuclear perimeter by day 11 (from 36.51 ± 3.50 to 30.35 ± 2.04), suggesting a more pronounced effect of this preparation on the regression of inflammatory processes or cellular changes. No changes in nuclear perimeter were observed in the control group during the examined intervals, confirming that the changes recorded in the therapeutic groups were a result of the applied treatments. Comparative analysis between the groups revealed statistically significant differences at all time points (*p* < 0.001). The Bonferroni test indicated that all therapeutic groups differed significantly from the control group (*p* < 0.001) at baseline and on day 1, while a significant difference was also observed between the standard basic therapy and the therapy with the addition of Propoherb G^®^ on day 11 after treatment.

Table 10 presents changes in nuclear Feret’s diameter before and after the applied therapy in the examined groups. In the therapeutic groups, a highly statistically significant reduction in Feret’s diameter was observed (Friedman test, *p* < 0.001), with a significant decrease already evident on day 1 after therapy, which continued through day 11. The most pronounced reduction was seen in the group receiving basic therapy with the addition of Propoherb G^®^ (13.30 → 11.77 → 10.98). Basic therapy alone also showed a significant change, although of slightly lower magnitude after 11 days. No changes were observed in the control group over time, with values remaining constant (10.16 ± 0.74), confirming that the observed changes in the experimental groups were due to the therapeutic interventions rather than the passage of time. Intergroup comparisons at all three time points revealed statistically significant differences (Kruskal–Wallis’s test, *p* < 0.001), confirming the effectiveness of the therapy, particularly when comparing the therapeutic groups with the control group (Mann–Whitney test, *p* < 0.001).

## 3. Discussion

In accordance with scientific advancements, our understanding of the etiology, pathogenesis, and clinical presentation of periodontal diseases is constantly evolving, and consequently, so is the concept of their treatment. Due to the invasive potential of microorganisms and their ability to penetrate periodontal tissues, basic therapy alone was once considered sufficient. However, antimicrobial therapy as an adjunct to mechanical treatment has gained significant attention in periodontal disease management. Oral biofilm is the main etiological factor in the development of gingivitis and periodontitis, making mechanical biofilm control the most effective approach for its elimination; nevertheless, this requires exceptional skill and precision. For these reasons, as well as several others, research has been conducted to find an adequate agent that could complement the effects of basic therapy. Numerous chemotherapeutic agents have been presented and studied in the literature, but most have shown undesirable effects when used over extended periods. The growing interest in a healthier lifestyle and environmental preservation has created a need to explore natural products, particularly those containing plant extracts [23].

Herbal products have been used for centuries to maintain oral hygiene and to treat gum diseases, such as cloves, turmeric, aloe vera, green tea, cinnamon, and others. They generally do not have adverse effects and are highly valued because they exhibit antimicrobial, antioxidant, antiseptic, and anti-inflammatory properties, and due to their anti-collagenase activity, they may potentially influence wound healing [23,24]. These products are preferred over conventional chemical agents not only for their biological activity but also for their safety and lower cost. Long-term use of conventional agents can lead to numerous drug-related adverse reactions, side effects, and resistance [25,26,27,28]. In recent years, there have been numerous attempts to test plants and their products to demonstrate their specific anti-biofilm effects, as they represent an attractive potential adjunct to mechanical, basic therapy. Phytotherapy can be applied in combination with conventional agents or alone, depending on the condition. Due to their numerous benefits, availability, and minimal adverse effects, herbal preparations represent an excellent option for controlling dental biofilm and the progression of periodontal diseases [29,30]. Overall, the application of basic periodontal therapy leads to an improvement in periodontal status, while the adjunctive use of Propoherb G^®^ demonstrates the greatest effectiveness in reducing the need for further periodontal treatment. Research in the field of herbal products is, however, still in its early stages.

The results of this study demonstrated the benefits of using the herbal preparation Propoherb G^®^ as an adjunct to basic therapy in the treatment of periodontal disease, measured through the clinical parameters such as the PLI (plaque index), BOP (bleeding on probing) and CAL (clinical attachment level). Improvements in these clinical parameters were observed over the study period, which can primarily be attributed to the basic therapy applied in both groups, considered the standard in periodontal treatment. A potential limitation of this study is the baseline demographic imbalance, specifically regarding the significant age difference between the treatment and control groups. This discrepancy was primarily due to the consecutive enrollment of available patients during the recruitment period, which resulted in a notably younger control group. Given that age is a well-established risk factor for periodontal disease, it is possible that this factor influenced the observed clinical outcomes. However, due to the limited sample size and the exploratory nature of the study, additional adjusted statistical analyses to account for age were not performed. Consequently, these findings should be interpreted with caution, and future research with age-matched cohorts is warranted to further validate our results. At the beginning of the study, the therapeutic groups had similar baseline values of the plaque index (approximately 1.8–1.9), whereas the control group showed a lower value (1.28). After the applied therapy, a significant reduction in the PLI was observed in the therapeutic groups (*p* < 0.001). The lowest value was recorded in the group receiving Propoherb G^®^ as an adjunct (0.19 ± 0.25), while in the group treated with basic therapy alone the index was 0.83 ± 0.37. No changes were observed in the control group (1.28 ± 0.50). These findings confirm the importance of the plaque index as a reliable clinical indicator of oral hygiene and treatment effectiveness, as well as the additional effect of the phytotherapeutic preparation Propoherb G^®^ in reducing dental plaque and gingival inflammation. In conclusion, the applied therapeutic modalities proved effective in reducing the plaque index, with Propoherb G^®^ showing beneficial effects as an adjunct to basic therapy compared with conventional basic therapy alone. A significant reduction in BOP was observed in the group receiving basic therapy combined with Propoherb G^®^ (0.16 ± 0.22), which was statistically significant compared to standard basic therapy (0.99 ± 0.36) (*p* < 0.001), as well as in relation to the control group (0.0 ± 0.0) (*p* < 0.001). Additionally, after therapy, statistically significant reductions in CAL were noted in the treated groups (*p* < 0.001), with the most pronounced improvement observed in the group receiving Propoherb G^®^ alongside basic therapy (2.14 ± 0.26). These findings are in line with numerous studies investigating herbal preparations used in various forms for the prevention and management of periodontal disease. Yaghini et al. conducted a randomized, double-blind, controlled study to assess clinical outcomes following subgingival application of an herbal gel (oak and coriander extract), demonstrating statistically significant improvements in periodontal clinical parameters [31]. Studies by Singh A. et al., Kaur H. et al., Siddharth M. et al., and Pérez-Pacheco C.G. et al. examined clinical parameters including PI, GI, BOP, PPD, and CAL in groups treated with basic therapy, herbal preparations, and chlorhexidine, showing significant improvements in BOP, PPD, and CAL, as well as reductions in inflammatory mediators when delivered into periodontal pockets, compared to the use of 0.2% CHX gel [32,33,34,35]. Bhat et al. reported that subgingival injection of aloe vera extract gel could improve periodontal health, suggesting that aloe vera extracts, due to their antioxidant properties, may be useful in preventing and treating periodontal disease [36]. Further studies by Mahyari S. et al., Vangipuram S. et al., Irfan M. et al., Sparabombe S. et al., Mangesh G. et al., and Kim Y.R. et al. investigated the effects of various plant-based mouth rinses, including turmeric, aloe vera, green tea, guava leaf extract, and coriander extract. These studies demonstrated the efficacy of such rinses in reducing PI, GI, and BOP, with significant improvements in overall periodontal health, all without reported adverse effects compared to chlorhexidine (CHX) [37,38,39,40,41,42].

Periodontopathogenic microflora comprises a group of Gram-negative anaerobic bacteria, among which *Aggregatibacter actinomycetemcomitans* (*A.a*.) and *Porphyromonas gingivalis* (*P.g.*) are particularly notable due to their aggressive potential. Their pathogenicity lies in their ability to directly and indirectly modify immune mechanisms in a destructive manner, which is a key factor in the development of periodontitis [43,44]. *A. actinomycetemcomitans* is a non-motile coccobacillus whose virulence factors include leukotoxin, cytotoxic toxins, immunosuppressive components, and inhibitors of polymorphonuclear cell function, which directly damage periodontal tissue and impair the host immune response [45,46]. *P. gingivalis* is a Gram-negative anaerobic bacillus possessing fimbriae and producing porphyrin pigment. This microorganism generates low-molecular-weight metabolites and toxic enzymes, including immunoglobulin-degrading proteases and gingipains, which cause local tissue destruction and disrupt immune responses through the induction of cytokines such as IL-6 and matrix metalloproteinases (MMPs) [45,47]. Although *A.a*. and *P.g*. utilize different pathogenic mechanisms, their ultimate effect is similar: osteoclast activation mediated by cytokines and prostanoids, including PGE_2_, TNF-α, IL-1β, IL-6, IL-17, and RANKL, contributing to periodontal tissue degradation and disease progression [48,49]. Given the importance of microorganisms in the etiology of periodontal diseases, the results of this study demonstrated that basic therapy significantly reduces the presence of periodontopathogenic bacteria (*Porphyromonas gingivalis*, *Aggregatibacter actinomycetemcomitans*, and *Treponema denticola*) over a short period (*P.g* = 23.30%, *A.a*. = 36.70%, *T.d*. = 43.30%), although this effect is temporary, with some bacteria showing reinfection within 11 days. In contrast, the combination of basic therapy with the herbal preparation Propoherb G^®^ resulted in a significantly more stable reduction or complete elimination of these bacteria. For *P.g.*, positivity decreased from 66.7% pre-therapy to 46.6% on the first day and was completely eliminated by the eleventh day (0%) (*p* < 0.001). For *A.a.,* a significant reduction was observed between pre-therapy and measurements on the eleventh day, as well as between the first and eleventh days (*p* < 0.001), achieving a higher eradication rate than basic therapy alone (*p* < 0.001). For *T.d*., positivity decreased from 76.7% to 50% on the first day (*p* = 0.018), and then dramatically to 6.7% on the eleventh day (*p* < 0.001), with a significant reduction between the first and eleventh days (*p* < 0.001). Intergroup comparisons showed that the addition of the herbal preparation was significantly more effective than basic therapy alone, while the control group showed no changes, confirming the therapeutic effect of the applied procedures. These findings suggest that phytotherapy can be an effective adjunct to basic therapy, enhancing the durability of the antibacterial effect and potentially reducing the risk of reinfection, which is important for improving long-term clinical outcomes in periodontitis treatment. Following phytotherapy, several studies reported microbiological outcomes demonstrating a significant reduction in the prevalence of periodontopathogenic microorganisms. Nashwah et al. showed, using qRT-PCR analysis, a reduction in the expression of genes associated with biofilm formation, indicating that frankincense extract exhibits antibacterial and antibiofilm activity against *P. gingivalis* and could be considered a potential therapeutic option for periodontitis [50]. Ayub et al. conducted a study investigating the antibacterial and antibiofilm properties of essential oils of cumin (*Cuminum cyminum*) and fennel (*Foeniculum vulgare*) on clinically isolated *P. gingivalis* and *Prevotella intermedia* obtained from periodontal pockets. Microbiological and molecular tests confirmed the antibacterial and antibiofilm activity of cumin and fennel oils against clinically isolated *P. gingivalis* and *P. intermedia* [51]. Research has also shown that cumin possesses significant antibiofilm potential and inhibits Gram-negative bacterial pathogens [52]. It should be emphasized that the studies employed different methods for collecting microbial samples, as well as various analytical techniques, such as cell culture, microscopy, and real-time PCR, which significantly complicates direct comparison of the results [53,54].

The basis of cytomorphometry lies in the analysis of cellular morphological characteristics, which allows for the assessment of their biological behavior and the identification of pathological changes [22,55]. The cell nucleus functions as the regulatory center of the cell, overseeing metabolic processes and monitoring morphological changes [56]. Cytological and morphological methods are used for the early detection of abnormalities, enabling the application of cytomorphometry in the assessment of gingival tissue inflammation [57]. The superficial layer of epithelial cells consists of desquamated polygonal cells of irregular shape, which can be easily sampled and microscopically analyzed to evaluate morphological effects, including the impact of therapy on gingival inflammation. Molecular-level changes directly influence cellular morphology; while the cytoplasm reflects normal cell function, the nucleus regulates its biological activity [58,59]. During cell differentiation, morphological and physiological changes occur, reflected in the size and structure of the nucleus [60]. The results of our study showed a significant reduction in nuclear area in the therapeutic groups after treatment (*p* < 0.001), with the greatest decrease observed in the group receiving Propoherb G^®^ (from 92.61 ± 24.91 μm^2^ to 62.96 ± 13.63 μm^2^). The nuclear perimeter decreased from 36.51 ± 3.50 μm to 30.35 ± 2.04 μm, and Feret’s diameter declined from 13.30 μm to 10.98 μm in the same group. Standard basic therapy also led to changes, though to a lesser extent, while the control group showed no significant alterations. Statistical analysis confirmed that differences between the therapeutic and control groups were significant across all measurement time points (baseline, day 1, and day 11; *p* < 0.001). The Bonferroni test further highlighted that the combination of basic therapy with Propoherb G^®^ achieved better results than basic therapy alone on day 11, indicating an additional effect of the phytotherapeutic preparation in reducing gingival inflammation. It is well known that the nuclei of cells in the stratified squamous gingival epithelium increase in size during inflammation, which is consistent with the findings of this study [60]. Obradović and colleagues [61] also reported a significant reduction in nuclear size following basic treatment of chronic periodontitis (*p* < 0.05), with an even more pronounced effect observed when low-level laser therapy (LLLT) was combined with basic therapy (LLLT + SRP) (*p* < 0.001). Since nuclei of stratified squamous epithelial cells typically enlarge during inflammation, these findings suggest that adjunctive LLLT enhances the therapeutic outcome in reducing inflammation compared to basic therapy alone. Similarly, Igic and colleagues [59] found that SRP treatment reduces the size of epithelial cell nuclei, although not to the level observed in healthy tissue. When adjunctive LLLT was applied, nuclear size approached that of healthy subjects, and the differences before and after treatment were statistically significant (*p* < 0.001). The results of our study indicate that adding adjunctive therapy to basic treatment leads to a more effective reduction of gingival tissue inflammation, which aligns with previous findings.

Data from the literature indicates that various herbal preparations, as well as the methods and timing of their application in the treatment of periodontitis, are consistent with the results of our study and show encouraging outcomes [62]. However, further research is needed to clarify the potential use of herbal preparations in the prevention and treatment of periodontal diseases. The combination of conventional periodontal therapy with phytotherapy has not yet been incorporated into standardized treatment protocols for patients with chronic periodontitis.

## 4. Materials and Methods

The randomized prospective clinical study was conducted at the Department of Periodontology and Oral Medicine of the Clinic of Dental Medicine, Faculty of Medicine, University of Nis, Serbia. The study was prepared in accordance with the principles of the Helsinki Declaration and approved by the competent Ethics Committee of the Faculty of Medicine, Nis (number: 12-3588-2/1) on 7 April 2022. This study was registered retrospectively with the clinical trial registration number ISRCTN10262904 (https://www.isrctn.com/ISRCTN10262904, the name of the registry: “Testing whether an herbal product can reduce gum inflammation when used alongside standard periodontal treatment”, accessed on 20 February 2026). The research included 90 systemically healthy participants between 30 and 60 years of age with a diagnosis of chronic periodontitis who met the following diagnostic criteria according to classification of periodontal and peri-implant diseases from 2018 [62]: gingival inflammation, the presence of periodontal pockets with a depth of 3.5 to 5.5 mm, clinical attachment loss from 2 mm to 4 mm (CAL), the presence of supra- and subgingival hard deposits, radiographic evidence of horizontal bone loss, and the presence of at least 10 teeth in each jaw. The destruction of periodontal structures was assessed based on clinical examination and orthopantomography. Exclusion criteria for the study were as follows: the presence of systemic or autoimmune diseases that affect the course of the condition, the use of antibiotics, anti-inflammatory drugs, or locally applied preparations in the last 3 months, mechanical or surgical periodontal therapy performed in the last 6 months, pregnancy and lactation, heavy smokers, participants younger than 18 years, participants with acute or chronic infections, or those who had undergone radiation or chemotherapy.

**Figure 1:**
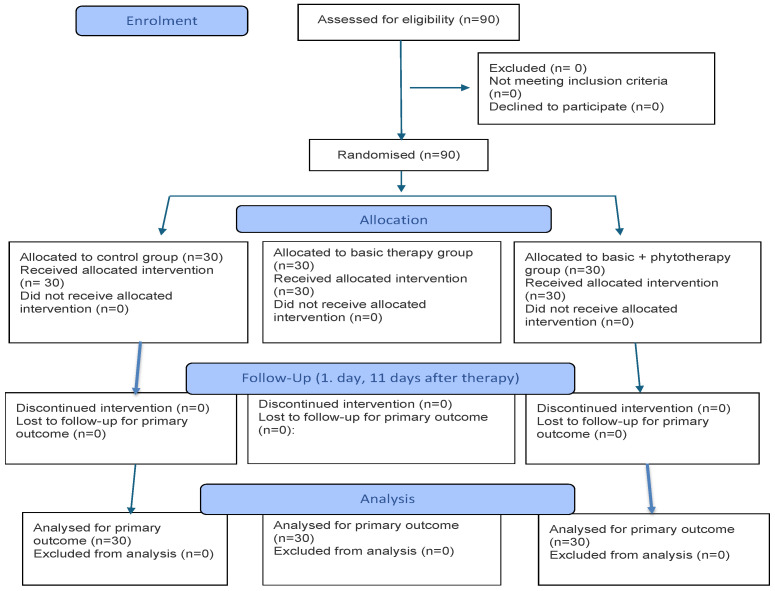
CONSORT flow diagram.

Before the start of the study, all participants were thoroughly informed about the procedures required for conducting the research, and only those who met the research criteria and provided written consent were included. After signing the consent form, all participants were randomly assigned to three groups: the control (placebo) group, consisting of subjects who had been treated for periodontitis one year or more before the beginning of the study and were considered to have a stable periodontal status, that is, clinically healthy periodontium, and who received physiological saline (n = 30). The control group was defined as a stable post-treatment group for ethical reasons, to avoid exposing participants with active periodontitis to a placebo. This approach allows comparison of the effects of adjunct therapy in a stable population. The therapeutic (basic therapy) group (Test 1) underwent periodontal therapy (n = 30), and in the phytotherapy group (Test 2), phytotherapy was used as an adjunct to mechanical treatment (SRP + Propoherb G^®^) (n = 30). Neither the participants nor the researchers who assigned them were aware of the group allocation prior to study enrollment. All clinical examinations and biological sample collections were performed by a single trained examiner who coded the participants and samples, while the personnel performing the microbiological analysis processed the samples under coded identifiers without knowledge of the group assignment (Figure 1).

Participants in the test groups were subjected to basic therapy, which included the identification and removal of oral biofilm and other dental deposits using polishing paste for removing soft deposits and rotating brushes, removal of supra- and subgingival calculus, and root surface instrumentation with an ultrasonic device (Woodpecker HW-1, UDS-J, Medical Instrument Company, Guilin, China). Treatment of the periodontal pocket was performed using periodontal curettes (Gracey 5/6 and 7/8, Chicago, IL, USA), followed by irrigation of the periodontal pocket contents with 3% hydrogen peroxide using a sterile single-use syringe (5 mL, Romed, Holland, Wilnis, The Netherlands) and a sterile needle (23 G, Nipro, Mechelen, Belgium) bent at an angle of approximately 110–115° relative to the longitudinal axis of the handle. Participants also received motivation and instruction for maintaining oral hygiene, as well as guidance on eliminating potential risk factors contributing to the accumulation of oral biofilm.

In the participants of the phytotherapy group, phytotherapy was performed after the mechanical treatment using the Propoherb G^®^ solution (Institute for Medicinal Plant Research “Dr Josif Pančić”, Belgrade, Serbia). The preparation was applied with a sterile disposable syringe (5 mL, Romed, Holland) and a sterile needle (23 G, Nipro, Belgium) bent at approximately 110–115° relative to the longitudinal axis of the handle, in a volume of 0.1 mL per periodontal pocket (subgingival application of the solution for each pocket lasted 1 min per quadrant, with isolation using sterile cotton rolls). The ingredients of Propoherb G^®^ are as follows: highly purified concentrated propolis extract (2%), a mixture of extracts of sage leaf, chamomile flower, and plantain leaf (20%), concentrated rosehip extract (2%), peppermint essential oil (0.2%), honey, polysorbate 20, potassium sorbate, and water. The therapy, lasting ten consecutive days, was performed by a single periodontist, and all participants received instructions for proper oral hygiene maintenance.

In the control group, physiological saline was applied using a sterile disposable syringe (5 mL, Romed, Holland, Wilnis, The Netherlands) and a sterile needle (23 G, Nipro, Mechelen, Belgium) bent at approximately 110–115° relative to the longitudinal axis of the handle, in a volume of 0.1 mL per secondary gingival sulcus for ten consecutive days. The therapy was completed in all participants, and no adverse effects were reported during the study.

### 4.1. Examination of Clinical Parameters

During the initial assessment and subsequent follow-up visits (after the ten-day treatment sessions), the Green–Vermilion plaque index (PLI), bleeding on probing (BOP), and clinical attachment level (CAL) were recorded using the following criteria.

The plaque index was assessed on the mesial, distal, buccal, and lingual surfaces of each tooth and scored as follows:

0—no plaque present;

1—plaque covering up to one-third of the tooth surface;

2—plaque covering more than one-third but less than two-thirds of the exposed tooth surface;

3—plaque covering more than two-thirds of the exposed tooth surface.

Gingival bleeding on probing (BOP) was assessed according to a three-point scoring system:

0—no bleeding on probing;

1—bleeding within 10 s of probing;

2—bleeding occurring between 10 and 20 s after probing;

3—bleeding detected immediately upon probing.

The clinical attachment level (CAL) was determined by measuring the distance from the base of the periodontal pocket to the cemento-enamel junction, expressed in millimeters, using a manual periodontal probe (Medesy, Maniago, Italy).

### 4.2. Polymerase Chain Reaction (PCR) Analysis

Subgingival specimens were obtained at baseline, after the first day, and after the treatment session to determine the levels of Aggregatibacter actinomycetemcomitans, Porphyromonas gingivalis, and Treponema denticola using PCR analysis. Sample collection was performed with sterile absorbent paper points (Dentplus, DiaDent Group International Inc., Cheongju, Republic of Korea), which were inserted into the deepest interproximal periodontal pocket within each dental quadrant. For every patient, samples from the deepest site in each quadrant (90 patients, 6 sites, 5 paper points per site) were collected to precise time points. After removal, each paper point was transferred into a sterile 1.5 mL Eppendorf tube containing 100 μL of sterile deionized water and immediately stored at −70 °C until DNA extraction.

Bacterial DNA extraction involved incubating the samples with 10% proteinase K (Thermo Fisher Scientific, Waltham, MA, USA) at 56 °C for 30 min, followed by enzyme inactivation at 94 °C for 15 min using a TS 100C thermoshaker (Biosan, Riga, Latvia). PCR amplification was carried out in a 25 μL reaction mixture composed of 2.5 μL PCR buffer, 2.5 mM MgCl_2_, 0.2 mmol/L dNTPs, 0.2 μM species-specific primers, 0.1 U DreamTaq DNA polymerase (Thermo Fisher Scientific™, Waltham, MA, USA), and 4 μL of extracted DNA. Amplification was performed in a thermal cycler (Peqlab, PeqSTAR, Erlangen, Germany) with the following program: initial denaturation at 95 °C for 3 min; 35 cycles of denaturation at 95 °C for 30 s, annealing at temperatures specific for each primer pair (Table 11) for 30 s, and extension at 72 °C for 45 s; followed by a final extension step of 72 °C for 5 min. The resulting PCR products were separated by electrophoresis on an 8% polyacrylamide gel, stained with ethidium bromide, and visualized under UV illumination using a UV Transilluminator (Fisher Scientific MA, USA).

### 4.3. Cytomorphometric Analysis

At the beginning of the study, after the first day, and at the end of therapy, gingival swabs were collected from all participants using sterile cotton swabs, and direct smears were prepared on microscope slides. The dried and fixed gingival smear preparations were then stained using the standard hematoxylin–eosin (HE) method. After completing the staining process, the slides were covered with cover slips and mounted using DPX mounting medium. These prepared slides were ready for microscopic analysis at high magnification. For morphometric analysis, the ImageJ software package, version 1.52a (Public Domain Software, Wayne Rasband, National Institutes of Health, Bethesda, MD, USA) was used. Microscopic examinations were performed with a Nikon Eclipse 50i microscope (Tokyo, Japan) using a ×40 objective. Color microphotographs were captured using a high-resolution digital camera (Nikon DS-Fi1, Tokyo, Japan) connected to the microscope. After acquisition, the images were transferred to a computer and converted to 8-bit resolution. Following calibration, the images were manually analyzed using a computer mouse.

During the analysis, the following nuclear parameters were evaluated: Nuclear area (μm^2^)—the surface area of the nucleus; Perimeter (μm)—the length of the nuclear outline; Feret diameter—the longest distance between any two points along the nuclear boundary.

### 4.4. Statistical Analysis

Data collection and organization were performed using Microsoft Excel, while statistical analysis was conducted with the IBM SPSS Statistics software package, version 22.0 (Chicago, IL, USA). The results of the statistical analysis were presented in tables. From the descriptive statistical indicators, standard parameters for qualitative and quantitative variables were used: absolute and relative frequencies, arithmetic mean ($\overset{-}{x}$), and standard deviation (SD). The normality of data distribution was tested using the Kolmogorov–Smirnov and Shapiro–Wilk tests. Depending on whether the conditions of normal distribution were met, the following methods were applied for comparison of numerical values between groups: in the case of normal distribution: one-way analysis of variance (ANOVA) with post hoc Bonferroni test; in the case of deviation from normality: the Kruskal–Wallis’s test, followed by a Mann–Whitney test for pairwise comparisons. For the analysis of parameters over time within the same groups (repeated measurements), the following tests were used: repeated-measures ANOVA when the data distribution was normal; Friedman test with post hoc Wilcoxon test when the distribution was not normal. To analyze the statistical significance of differences in absolute frequencies, the Chi-square test was used, while for related samples, Cochran’s Q test was applied, followed by the McNemar test for pairwise comparisons. Statistical hypotheses were tested at a significance level of α = 0.05. Results with *p* < 0.05 were considered statistically significant.

## 5. Conclusions

Based on the obtained results, it can be concluded that the use of the herbal preparation Propoherb G^®^ as an adjunct to basic therapy leads to a statistically significant improvement in PLI, BOP and CAL values over the observed period, as well as a marked reduction in the tested periodontopathogens (*Aggregatibacter actinomycetemcomitans*, *Porphyromonas gingivalis*, and *Treponema denticola*) and an improvement in the assessed cytomorphometric parameters. The use of an herbal preparation could yield better clinical outcomes when combined with conventional treatment methods. However, further studies involving a larger number of participants and longer observation periods are needed to determine the most appropriate method of application. Future research should aim to develop standardized protocols to identify the effects of different herbal products used in dentistry.

## Figures and Tables

**Table 1:** Gender and age distribution of the examined group.

		Test 1	Test 2	Control Group
Gender ^1^	Men n (%)	11 (36.7%)	6 (20.0%)	3 (10.0%)
	Women n (%)	19 (63.3%)	24 (80.0%)	27 (90.0%)
Age ^2^ mean ± SD		39.70 ± 7.14	43.50 ± 5.65	26.47 ± 1.85 ^a,b^

^1^ Chi-square test; ^2^ ANOVA; ^a,b^ (Bonferroni test): ^a^ Basic therapy vs. control; ^b^ basic therapy + Propoherb G^®^ vs. control.

**Table 2:** Plaque index—PLI before and after therapy between test groups.

	Test 1	Test 2^®^	Control Group	*p* ^3^
Before therapy	1.93 ± 0.56	1.83 ± 0.51	1.28 ± 0.50	<0.001 ^a,b^
After therapy	0.83 ± 0.37	0.19 ± 0.25	1.28 ± 0.50	<0.001 ^a,b,c^
*p* ^4^	<0.001	<0.001	/	

^3^ Kruskal–Wallis’s test; ^4^ Wilcoxon test. ^a,b,c^ (Mann–Whitney test); ^a^ basic therapy vs. control; ^b^ basic therapy + Propoherb G^®^ vs. control; ^c^ basic therapy vs. basic therapy + Propoherb G^®^.

**Table 3:** Bleeding on probing index—BOP before and after therapy by study group.

	Test 1	Test 2	Control Group	*p* ^3^
Before therapy mean ± SD	1.58 ± 0.47	1.65 ± 0.44	0.0 ± 0.0	<0.001 ^a,b^
After therapy mean ± SD	0.99 ± 0.36	0.16 ± 0.22	0.0 ± 0.0	<0.001 ^a,b,c^
*p* ^4^	<0.001	<0.001	/	

^3^ Kruskal–Wallis’s test; ^4^ Wilcoxon test. ^a,b,c^ (Mann–Whitney); ^a^ Basic therapy vs. control; ^b^ basic therapy + Propoherb^®^ vs. control; ^c^ basic therapy + Propoherb^®^ vs. basic therapy.

**Table 4:** CAL values before and after therapy in the examined groups.

	Test 1	Test 2	Control Group	*p* ^3^
Before therapy mean ± SD	2.96 ± 0.26	3.10 ± 0.18	0.8 ± 0.33	<0.001 ^a,b^
After therapy mean ± SD	2.47 ± 0.22	2.14 ± 0.26	0.8 ± 0.33	<0.001 ^a,b,c^
*p* ^4^	<0.001	<0.001	/	

^3^ Kruskal–Wallis’s test; ^4^ Wilcoxon test. ^a,b,c^ (Mann–Whitney); ^a^ Basic therapy vs. control; ^b^ basic therapy + Propoherb G^®^ vs. control; ^c^ basic therapy + Propoherb G^®^ vs. basic therapy.

**Table 5:** Positivity for *Porphyromonas gingivalis* before and after therapy in the study groups.

Group	Before Therapy	1st Day After Therapy	11th Day After Therapy	*p* ^5^
Test 1 n, (%)	18 (60.70%)	7 (23.30%) ^a^	16 (53.30%) ^c^	<0.001
Test 2 n, (%)	20 (66.70%)	14 (46.62%) ^a^	0 (0.00%) ^b,c^	<0.001
Control group	3 (10.00%)	3 (10.00%)	3 (10.00%)	/
*p* ^1^	0.010	0.330	<0.001	

^5^ Cochran’s Q test; ^a,b,c^ (Mc Nemar test); ^a^ before therapy vs. 1 day after therapy; ^b^ before therapy vs. 11 days after therapy; ^c^ 1 day after therapy vs. 11 days after therapy. ^1^ Chi-square test.

**Table 6:** Positivity for *Aggregatibacter actinomycetemcomitans* before and after therapy in the study groups.

Group	Before Therapy	1st Day After Therapy	11th Day After Therapy	*p* ^5^
Test 1 n, (%)	17 (56.70%)	11 (36.70%) ^a^	15 (50.00%) ^c^	0.009
Test 2 n, (%)	19 (63.30%)	15 (50.00%) ^a^	1 (3.30%) ^b,c^	<0.001
Control group n, (%)	3 (10.00%)	3 (10.05)	3 (10.00%)	/
*p* ^1^	<0.001	0.008	<0.001	

^5^ Cochranov Q test; ^a,b,c^ (Mc Nemar test); ^a^ before therapy vs. 1 day after therapy; ^b^ before therapy vs. 11 days after therapy; ^c^ 1 day after therapy vs. 11 days after therapy. ^1^ Chi-square test.

**Table 7:** Positivity of *Treponema denticola* before and after therapy in the study groups.

Group	Before Therapy	1st Day After Therapy	11th Day After Therapy	*p* ^5^
Test 1 n, (%)	24 (80.00%)	13 (43.30%) ^a^	20 (66.70%) ^c^	<0.001
Test 2 n, (%)	23 (76.70%)	15 (50.00%) ^a^	2 (6.70%) ^b,c^	<0.001
Control group n, (%)	2 (6.70%)	2 (6.70%)	2 (6,70%)	/
*p* ^1^	<0.001	0.002	<0.001	

^5^ Cochranov Q test; ^a,b,c^ (Mc Nemar test); ^a^ before therapy vs. 1 day after therapy; ^b^ before therapy vs. 11 days after therapy; ^c^ 1 day after therapy vs. 11 days after therapy. ^1^ Chi-square test.

**Table 8:** Nuclear area (surface) before and after the therapy in the examined groups.

Group	Before Therapy	1 Day After Therapy	11 Days After Therapy	*p* ^6^
Test 1	92.25 ± 24.11	73.73 ± 17.70 ^a^	77.35 ± 16.68 ^b,c^	<0.001
Test 2	92.61 ± 24.91	80.15 ± 19.31 ^a^	62.96 ± 13.63 ^b,c^	<0.001
Control group	58.21 ± 5.28 ^d,e^	58.21 ± 5.28 ^d,e^	58.21 ± 5.28 ^d,e^	/
*p* ^3^	<0.001	<0.001	<0.001	

^6^ Friedman test; ^a,b,c^ (Wilcoxon Test): ^a^ before therapy vs. first day after therapy; ^b^ before therapy vs. 11th day after therapy; ^c^ first day after therapy vs. 11th day after therapy. ^3^ Kruskal–Wallis’s test; ^d,e^ (Mann–Whitney test): ^d^ control vs. test 1, ^e^ control vs. test 2.

**Table 9:** Nuclear perimeter before and after therapy in the examined groups.

Group	Before Therapy	1 Day After Therapy	11 Days After Therapy	*p* ^7^
Test 1	35.57 ± 4.46	31.90 ± 3.57 ^a^	32.77 ± 3.48 ^b,c^	<0.001
Test 2	36.51 ± 3.50	32.60 ± 3.36 ^a^	30.35 ± 2.04 ^b,c^	<0.001
Control group	28.71 ± 1.43	28.71 ± 1.43	28.71 ± 1.43	/
*p* ^2^	<0.001 ^d,e,f^	<0.001 ^d,e,f^	<0.001 ^d,f^	

^7^ ANOVA; ^a,b,c^ (*t*-test): ^a^ before therapy vs. first day after therapy; ^b^ before therapy vs. 11th day after therapy; ^c^ first day after therapy vs. 11th day after therapy. ^2^ ANOVA; ^d,e,f^ (Bonferroni test): ^d^ test 1 vs. control; ^e^ test 2 vs. control; ^f^ test 1 vs. test 2.

**Table 10:** Feret’s diameter before and after the applied therapy in the examined groups.

Group	Before Therapy	1 Day After Therapy	11 Days After Therapy	*p* ^6^
Test 1	12.89 ± 1.47	11.58 ± 1.37 ^a^	11.85 ± 1.24 ^b,c^	<0.001
Test 2	13.30 ± 1.26	11.77 ± 1.23 ^a^	10.98 ± 0.76 ^b,c^	<0.001
Control group	10.16 ± 0.74 ^d,e^	10.16 ± 0.74 ^d,e^	10.16 ± 0.74 ^d,e^	/
*p* ^3^	<0.001	<0.001	<0.001	

^6^ Friedman test; ^a,b,c^ (Wilcoxon Test): ^a^ before therapy vs. first day after therapy; ^b^ before therapy vs. 11th day after therapy; ^c^ first day after therapy vs. 11th day after therapy. ^3^ Kruskal–Wallis’s test; ^d,e^ (Mann–Whitney test): ^d^ test 1 vs. control; ^e^ test 2 vs. control.

**Table 11:** Primer sequences and PCR parameters used for bacterial DNA amplification.

Bacterial Species	Primer	DNA Base Pairs	Temperature
*Porphyromonas gingivalis*	PGF	5′-AGGCAGCTTGCCATACTGCG-3′	55 °C
(*Pg*)	PGR	5′-ACTGTTAGCAACTACCGATGT-3′	
*Aggregatibacter*	Aa1F	5′-GCTAATACCGCGTAGAGTCGG-3′	55 °C
*actinomycetemcomitans* (*Aa*)	Aa1R	5′-ATTTCACACCTCACTTAAAGGT-3′	
*Treponema denticola*	TDF	5′-TAATACCGAATGTGCTCATTTACAT-3′	60 °C
(*Td*)	TDR	5′-TCAAAGAAGCATTCCCTCTTCTTCTTA-3′	

## Data Availability

The data that support the findings of this study are available from [Radmila Obradovic and Ivana Stankovic] but restrictions apply to the availability of these data, which were used under license for the current study, and so are not publicly available. Data are, however, available from the authors upon reasonable request and with permission of [Radmila Obradovic and Ivana Stankovic].

## Abbreviations

The following abbreviations are used in this manuscript:

*P.g.*	*Porphyromonas gingivalis*
*A.a.*	*Aggregatibacter actinomycetemcomitans*
*T.d.*	*Treponema denticola*
PLI	Plaque index
BOP	Bleeding on probing
CAL	Clinical attachment level
SRP	Scaling and root planning
GI	Gingival index
PPD	Periodontal pocket depth
CHX	Chlorhexidine
PGE_2_	Prostaglandin E_2_
TNF-α	Tumor Necrosis Factor α
IL-1β	Interleukin-1β
IL-6	Interleukin-6
IL-17	Interleukin-17
RANKL	Receptor Activator of Nuclear factor κB Ligand
PCR	Polymerase Chain Reaction
qRT-PCR	quality Real Time Polymerase Chain Reaction
DNA	Deoxyribonucleic acid
HE	Hematoxylin–Eosin
DPX	Dibutylphthalate Polystyrene Xylene
SD	Standard Deviation
ANOVA	Analysis of Variance
LLLT	Low-level laser therapy
